# Healthcare Professionals’ Perception Regarding Patient Rights and Safety in Najran, Saudi Arabia

**DOI:** 10.7759/cureus.50637

**Published:** 2023-12-16

**Authors:** Dawood Alyami, Ibraheem S Banihameem, Mohammed H Al-Mansour, Ali S AlRashah, Manassar Z Alsulieman, Hussain G Alsaqour, Mohammed S Alsagoor, Abdullah H Alshahi, Mahdi M Alyami, Ali R Alyami, Faisal H Alsharif, Amro M Mahmoud

**Affiliations:** 1 Department of Pharmacy, Khobash General Hospital, Najran, SAU; 2 Department of Administration, General Directorate of Health Affairs, Najran, SAU; 3 Department of Administration, Khobash General Hospital, Najran, SAU; 4 Department of Pharmacy, General Directorate of Health Affairs, Najran, SAU; 5 Department of Sociology, General Directorate of Health Affairs, Najran, SAU; 6 Department of Dentistry, Khobash General Hospital, Najran, SAU; 7 Department of Dentistry, Eradah Complex for Mental Health, Najran, SAU; 8 Department of Physiotherapy, General Directorate of Health Affairs, Najran, SAU; 9 Department of Dermatology, Khobash General Hospital, Najran, SAU

**Keywords:** saudi arabia, perception, safety patient, patient safety culture, patient rights

## Abstract

Background: Patient safety and rights are the most crucial aspects of healthcare quality. In Saudi Arabia, there is a paucity of evidence concerning the perspectives of healthcare providers on the rights and safety of patients. Hence, this study aimed to assess the perceptions of healthcare providers toward patients’ rights and safety in Najran, Saudi Arabia.

Methodology: A cross-sectional study was undertaken in Najran, Saudi Arabia, from December 2022 to October 2023, utilizing an online survey. This study included 307 healthcare providers who responded to the questionnaire via Google web link (Google LLC, Mountain View, California, United States).

Results: The present research comprised 307 healthcare personnel, of whom 65.8% were male and possessed a variety of academic backgrounds. The participants exhibited a high level of support for patients' rights, as evidenced by their agreement with 88.4-90% of questions on a variety of dimensions; this demonstrated their dedication to providing patient-centered care. Concerning medical errors, a significant proportion of respondents (74.9-86.1%) exhibited comprehensive comprehension and a readiness to disclose such incidents. Diverse viewpoints surfaced regarding the attribution of errors, the necessity of reporting, and the accountability for disclosure. The interdependence of patient rights and attitudes towards patient safety was highlighted by substantial positive correlations.

Conclusion: The viewpoints of healthcare professionals regarding medical errors and patients' liberties were discussed in this study. Advocacy for patients' rights is indicative of a commitment to patient-centered care that prioritizes autonomy and transparency. Although most participants demonstrated a willingness to report medical errors and possess a solid comprehension of their causes, divergent views emerged regarding attribution and disclosure. The interrelation between patient rights and attitudes toward patient safety was supported by positive correlations. The significance of continuous education in healthcare to promote a safety culture and enhance patient-centric practices is underscored by these results. Future research is needed to investigate the effects of culturally tailored interventions on the attitudes and practices of healthcare providers in Najran with regard to patient rights and safety.

## Introduction

Patient safety and rights are crucial concerns in healthcare quality. Safety is an essential element of quality management and a fundamental principle of patient care [1]. In the same way, it encompasses a multitude of activities pertaining to the enhancement of performance, management of risks and environmental safety, infection prevention, proper utilization of pharmaceuticals, security of instruments, secure clinical procedures, and the provision of a secure environment for care [1]. The healthcare system relies heavily on patient rights and safety, which significantly impact the satisfaction and overall well-being of persons seeking medical treatment [1]. Both facets are crucial for fostering trust and confidence between healthcare practitioners and patients, supporting the provision of exceptional care, and upholding ethical standards across the healthcare system [1]. Ensuring patient safety and upholding patient rights are fundamental values that form the basis of delivering healthcare that is of excellent quality, ethical, and centered upon the needs of the patient. By prioritizing these characteristics, not only do individual patients benefit, but it also enhances the general effectiveness and legitimacy of the healthcare system [1,2].

Healthcare institutions acknowledge patient safety as a critical component of their organizational culture, which constitutes a fundamental obligation for all healthcare practitioners. Ensuring patient safety is a crucial aspect of the organizational culture inside healthcare institutions, and it is a significant issue for all healthcare providers. Nevertheless, healthcare is intricate, and many factors affect its outcome [2]. According to the Institute of Medicine, patient safety is defined as “the prevention of harm to patients" [3]. The focal point is the care delivery system that is characterized by error prevention, knowledge acquisition from errors that do transpire, and the establishment of a safety-oriented culture that engages healthcare institutions, providers, and patients [3]. One-fifth of the public is exposed to medical errors, and this rate could be as high as 35-42% in the community [4]. Therefore, thousands of individuals could die or sustain injuries due to errors that are avoidable. Given the repercussions of medical errors, more research pertaining to patient safety is required [4].

Access to comprehensive data regarding the magnitude and specifics of hospital error rates associated with adverse patient incidents in Saudi Arabia is limited. However, it is claimed that approximately 40,000 medical error complaints are filed annually in Saudi Arabia and that medicolegal committees are notified of over 3000 instances of medical malpractice [5]. A previous study in Saudi Arabia reported that 20% of errors were linked with operating rooms and 18% were linked with emergency departments out of a total of 642 adverse events [6]. Evidence indicates that the prevalence of medication errors is also high in Saudi hospitals, ranging between 13 and 56 per 100 medication orders [7]. In addition, only 9% of Saudi hospitals were equipped with a medication safety officer, while a mere 30% had a medication safety unit.

Patient safety is acknowledged on a global scale as an essential healthcare concern [4]. Evidence indicates that a significant number of patients die annually in hospitals due to breaches in patient safety protocols [4]. In healthcare settings, improving patient safety remains a challenge as compared to other industries with highly reliable processes [3]. There is a scarcity of studies that examine the theoretical underpinnings of implementing patient safety systems in healthcare environments [3]. Similarly, the safety and rights of patients have emerged as a subject of national significance within the medical field. Healthcare professionals fulfill an essential responsibility as guardians of the rights and safety of patients [4]. This entails guaranteeing patients' timely access to hospital care, medications, and treatment, among other responsibilities [4]. The quality of patient care outcomes and critical determinants that are likely to have the greatest impact on error rates are the safety attitudes of healthcare professionals [8]. Evidence regarding the perception of healthcare providers regarding patients’ rights and safety is limited in Saudi Arabia. This evidence is crucial for policymakers to develop strategies to improve patient safety and health outcomes. Therefore, we aimed to assess the perceptions of healthcare providers toward patient rights and safety in Najran, Saudi Arabia.

## Materials and methods

This was an online cross-sectional study conducted from December 2022 to October 2023 among healthcare professionals in Najran, Saudi Arabia. This non-invasive investigation was after securing approval from the Central Institutional Review Board, Ministry of Health, Saudi Arabia (approval number: 22-67M). The study was carried out in accordance with the principles outlined in the Declaration of Helsinki.

Inclusion and exclusion criteria

Participants must be Saudi nationals presently employed in a healthcare setting in Najran and willing to voluntarily participate to be eligible for this research. Expatriates employed in Najran were included; however, individuals who solely responded to the sociodemographic questions were excluded from our study.

Data collection

A 32-item questionnaire was adapted for the survey from previous studies [9,10]. The questionnaire consisted of questions related to demographics (age, gender, education, profession, and work area) and general questions regarding perceptions of patients’ rights and safety. The types of questions included Likert scales and boxes with free text.

The recruitment process for survey participants involved the use of purposive sampling, which specifically targeted healthcare personnel residing in the Najran region of Saudi Arabia. The selection of Najran was predicated on the geographic region's healthcare diversity and the significance of gaining a comprehension of the viewpoints held by healthcare professionals there. The recruitment process entailed contacting diverse healthcare facilities, such as hospitals, clinics, and healthcare organizations located in Najran. Healthcare workers were invited through professional networks and direct communication with healthcare institutions. They were provided with a link to the online survey via Google Forms (Google LLC, Mountain View, California, United States).

After completing the research questionnaire, participants were requested to distribute the invitations to their colleagues. Additionally, it was requested that every participant distribute the survey to their email lists and social media profiles, such as Facebook (Meta Platforms, Inc., Menlo Park, California, United States), Twitter (X Corp., San Francisco, California, United States) and WhatsApp (Meta Platforms, Inc.). The study objectives were presented to the participants on the initial page of the questionnaire, adjacent to the assent statement, to furnish them with overarching information. The act of completing the survey was considered implicit consent. Multiple submissions were prevented in the Google Form by restricting responses to one per individual per submission. Additional text was included on the survey's summary page: "Should you have previously completed this survey, there is no necessity to resubmit it." This was done to mitigate the occurrence of duplicate responses from a single healthcare professional. A comprehensive assessment was conducted to determine the eligibility and conclusion of every returned questionnaire. 

For the protection of participants' privacy, the survey was intentionally structured to ensure complete anonymity. There was no collection of personally identifiable information, including names, addresses, or contact information. By default, Google Forms does not gather IP addresses or any other identifying information from respondents. To uphold confidentiality and safeguard data, survey responses were securely stored on servers that were password-protected and had restricted access, and only the research team had access to the gathered information.

Sample size and statistical analysis

To collect responses from healthcare professionals in the Najran region, a convenience sampling method was used for this study. The decision to use convenience sampling was based on the practical limitations of efficiently and promptly reaching a varied group of healthcare practitioners. Due to the survey being conducted online, a wide range of participants could be reached and this made it easier to get replies from persons who were easily accessible and ready to take part. The baseline characteristics of the participants were descriptively presented. The Kolmogorov-Smirnov test was employed to assess normality, given that the sample size exceeded 50 participants. A p-value below 0.05 was deemed to indicate statistical significance. Data analysis was carried out using IBM SPSS Statistics for Windows, Version 24.0 (IBM Corp., Armonk, New York, United States) and Microsoft Office Excel 2019 (Microsoft Corporation, Redmond, Washington, United States).

## Results

A survey instrument was distributed to 390 healthcare professionals and a total of 307 healthcare professionals took part in the research. The gender composition of the participants indicated that males comprised the majority (65.8%), while females comprised the remaining 33.2% (Table 1). In terms of professional credentials, the participants exhibited diversity in their academic backgrounds; the majority possessed a bachelor's degree (58.4%), while 14.2% held a master's degree and 12.9% held a diploma. A lesser percentage of the participants possessed a Ph.D. (9.4%) (Table 1). With regard to their place of employment, a minority of the participants (1.2%) were affiliated with dental clinics, whereas hospitals constituted the majority of the work areas (81.6%). Primary healthcare settings constituted 10.3% of the reported work areas, while other work areas were cited by 6.9% of the participants. The study's participants exhibited a wide spectrum of ages. The age group of 31-40 years comprised the largest proportion (38.4%), followed by the age group of 21-30 (29.7%). The age group of 41-50 years accounted for 17.9% of the sample. In contrast, the age groups of 61-70 years, 71-80 years, and less than 20 years constituted a reduced proportion of the overall distribution (Table 1).

**Table 1:** Characteristics of participants

Variable	n (%)
Gender	
Female	103 (33.2)
Male	204 (65.8)
Profession	
Nurse	128 (41.3)
Physician	102 (32.9)
Pharmacist	14 (4.5)
Radiology service staff	14 (4.5)
Social services	7 (2.3)
Laboratory staff	7 (2.3)
Others	38 (12.0)
Qualification	
Diploma	40 (12.9)
Bachelors	181 (58.4)
Masters	44 (14.2)
Doctor of Philosophy (PhD)	29 (9.4)
Others	16 (5.1)
Work area	
Hospital	253 (81.6)
Dental clinic	4 (1.2)
Primary healthcare	32 (10.3)
Others	21 (6.9)
Age	
Less than 20 years	4 (1.2)
21-30 years	92 (29.7)
31-40 years	119 (38.4)
41-50 years	56 (17.9)
51-60 years	11 (3.5)
61-70 years	2 (0.6)
71-80 years	3 (0.9)

Participants’ perceptions about patients’ rights

A significant majority of respondents (88.4%) concurred that patients are entitled to health-promoting and preventive healthcare services (Table 2). The consensus among the participants (88.1%) was that patients are entitled to compassionate, amicable, and friendly healthcare; this finding underscored the criticality of adopting a patient-centered approach. Furthermore, a significant percentage (88.7%) recognized the entitlement of patients to be attended to in an environment that is free from disturbance, clean, and comfortable. A substantial proportion of the participants (86.4%) confirmed that individuals with conditions have the entitlement to obtain all essential healthcare services. The consensus among the participants was that patients ought to be provided services without regard to their race, religion, sex, or social standing (86.5%). This highlights the dedication to cultivating a healthcare setting that places importance on fairness and inclusiveness. A significant majority of respondents (90%) agreed that patients are entitled to receive healthcare services in a secure environment, thereby underscoring the paramount importance of patient safety in healthcare provision. Furthermore, 86.8% of respondents agreed that patients and/or their family members/relatives have a legitimate expectation to be apprised of their health condition. This finding emphasizes the criticality of open and transparent communication. The participants (87.1%) demonstrated consensus regarding the proposition that patients should be permitted to accept or decline visitors in adherence to hospital policies; this acknowledges the importance of safeguarding patients' autonomy when it comes to arranging social interactions (Table 2). Moreover, a significant percentage (82.6%) recognized that patients have the legal entitlement to request a companion in accordance with hospital regulations. The majority of respondents (85.6%) acknowledged that patients have the right to file a complaint if their rights are violated (Table 2). 

**Table 2:** Participants’ perceptions about patients’ rights

Questions	Strongly Agree	Agree	Neutral	Disagree	Strongly Disagree
	n (%)	n (%)	n (%)	n (%)	n (%)
Patients have the right to access to health-promoting and preventive health services	79 (25.5)	195 (62.9)	30 (9.7)	1 (0.3)	2 (0.6)
Patients have the right to receive friendly, courteous, compassionate healthcare	91 (29.4)	182 (58.7)	23 (7.4)	10 (3.2)	1 (0.3)
Patients have the right to receive healthcare in a hygienic, comfortable, and noise-free environment	105 (33.9)	170 (54.8)	29 (9.4)	3 (1.0)	1 (0.3)
Patients has the right to access to all health care services required for their conditions	103 (33.2)	165 (53.2)	30 (9.7)	9 (2.9)	1 (0.3)
Patient should receive service regardless of his/her race, religion, sex, or social status	132 (42.6)	136 (43.9)	22 (7.1)	13 (4.2)	4 (1.3)
Patient has the right to receive health care services in a safe environment	129 (41.6)	150 (48.4)	18 (5.8)	4 (1.3)	3 (1.0)
Patients and/or family/relatives have the right to be informed about their health status	95 (30.6)	174 (56.1)	26 (8.4)	9 (2.9)	3 (1.0)
Patients have the right to accept or refuse visitors in accordance with the regulations of the hospital	86 (27.7)	184 (59.4)	27 (8.7)	9 (2.9)	1 (0.3)
Patients have the right to request a companion according to the rules and regulations of the hospital	92 (29.7)	164 (52.9)	42 (13.5)	6 (1.9)	2 (0.6)
Patients have the right to complain in case of violation of patient rights	93 (30.0)	173 (55.8)	38 (12.3)	3 (1.0)	1 (0.3)
Patients have the right to accept or refuse treatment procedures, medication, or hospitalization	95 (30.6)	168 (54.2)	34 (11.0)	8 (2.6)	2 (0.6)
Patients have the right to obtain information related to their health status, verbally or in writing	95 (30.6)	171 (55.2)	33 (10.6)	5 (1.6)	3 (1.0)
In any medical intervention, patient's consent must be obtained	107 (34.5)	156 (50.3)	40 (12.9)	4 (1.3)	1 (0.3)

Participants’ perceptions of medical errors

A significant proportion of the participants (84.9%) demonstrated a comprehensive comprehension of the factors contributing to medical errors, with the majority expressing a firm understanding (Table 3). Furthermore, a considerable percentage (86.1%) held the belief that their medical education had furnished them with the necessary skills to avert medical errors. Most of the participants (74.9%) exhibited a readiness to disclose errors committed by others, irrespective of the gravity of the consequences for the patient (Table 3). 

**Table 3:** Participants’ perceptions about medical errors

Questions	Strongly Agree	Agree	Neutral	Disagree	Strongly Disagree
	n (%)	n (%)	n (%)	n (%)	n (%)
I am trained and do understand the causes of medical errors	82 (26.5)	181 (58.4)	38 (12.4)	3 (1.0)	2 (0.6)
I have a good understanding of patient safety issues as a result of my medical training	92 (29.7)	183 (59.0)	25 (8.1)	6 (1.9)	1 (0.3)
My training is preparing me to prevent medical errors	93 (30.0)	174 (56.1)	31 (10.0)	6 (1.9)	3 (1.0)
I would feel comfortable reporting any errors other people had made, no matter how serious the outcome had been for the patient	74 (23.9)	158 (51.0)	55 (17.7)	18 (5.8)	3 (1.0)
A true professional does not make mistakes or errors	39 (12.6)	126 (40.6)	68 (21.9)	63 (20.3)	12 (3.9)
Human error is inevitable	54 (17.4)	146 (47.1)	80 (25.8)	19 (6.1)	8 (2.6)
Most medical errors result from careless nurses	19 (6.1)	84 (27.1)	79 (25.5)	86 (27.7)	37 (11.9)
Most medical errors result from careless doctors	29 (9.4)	80 (25.8)	98 (31.6)	77 (24.8)	23 (7.4)
Medical errors are a sign of incompetence	36 (11.6)	118 (38.1)	95 (30.6)	46 (14.8)	13 (4.2)
It is not necessary to report errors which do not result in adverse outcomes (harm) for the patient	26 (8.4)	85 (27.4)	52 (16.8)	105 (33.9)	38 (12.3)
All medical errors should be reported	98 (31.6)	166 (53.5)	33 (10.6)	8 (2.6)	3 (1.0)
Doctors have a responsibility to disclose errors to patients only if they result in patient harm	41 (13.2)	128 (41.3)	66 (21.3)	55 (17.7)	16 (5.2)
Patients have an important role in preventing medical errors	46 (14.8)	161 (51.9)	58 (18.7)	26 (8.4)	14 (4.5)

The correlation between each dimension of the ATPR (attitude toward patient rights) and the ATPS (attitude toward patient safety) including patient safety training, error reporting confidence, error inevitability, and disclosure responsibility was found to be statistically significant and positive, as shown in Table 4. The dimension of ATPS, working hours as error cause, showed a significant positive association with the ATPR dimension of patients’ communication and getting information rights. Moreover, in the ATPS dimension, patients’ involvement in reducing error was significantly associated with each of the ATPR dimensions except patients’ rights in management services.

**Table 4:** Correlation Analysis among Dimensions of ATPS and ATPR (N = 307) ATPS, attitude toward patient safety; ATPR, attitude toward patient rights *Not significant; ** Significant

Dimension of the ATPS	Dimension of the ATPR							
	The general attitudes towards patient rights		The attitudes towards including patients’ communication and getting information rights		The attitudes toward patient rights in management services		The attitudes toward patient rights in medical services	
	r	p	r	p	r	p	r	p
Patient safety training received	.49	.000**	.507	.000**	.45	.000**	.49	.000**
Error reporting confidence	.31	.000**	.32	.000**	.32	.000**	.33^*^	.000**
Working hours as error cause	.00	.934	.15	.011*	.05	.369	.05	.311
Error inevitability	.19	.001**	.22	.000**	.23	.000**	.19	.001**
Professional incompetence as error cause	.08	.158	-.02	.637	.01	.858	.03	.576
Disclosure responsibility	.28	.000**	.14	.016*	.27	.000**	.19	.001**
Patient involvement in reducing error	.16	.006**	.18	.001**	.07	.226	.16	.006**

## Discussion

The results of the study provide valuable insights into the viewpoints of healthcare practitioners regarding medical errors and the rights of patients. The strong consensus among patients regarding their rights, with strong agreement across multiple dimensions, demonstrates a praiseworthy dedication to providing care that is centred around the patient. This is consistent with the tenets of patient-centered care, which prioritize the provision of comprehensive healthcare services, autonomy, and transparency [11-13].

Conversely, the intricate nuances surrounding medical errors create a multifaceted landscape. Although many respondents exhibited a comprehensive comprehension of error causes and indicated a readiness to report errors, divergent viewpoints surfaced regarding attribution and disclosure. This intricacy reflects the prevailing body of literature, which frequently highlights the diverse and complex perspectives of healthcare practitioners regarding errors [14-17].

The observed positive correlations between patient rights and attitudes toward patient safety underscore the critical interdependence that exists between these two dimensions. This dynamic implies that an endorsement and acknowledgment of patients' rights are inextricably linked with a positive attitude toward patient safety. This also reflects the worldwide support from organizations like the World Health Organization for the integration of patient safety measures into the education and practices of healthcare professionals [18], and is in alignment with research that underscores the mutually beneficial connection between patient safety and the standard of healthcare as a whole [19, 20].

The implications of the study for patient-centered care are significant. The explicit recognition of patients' rights implies that healthcare practitioners understand the critical importance of patient autonomy and open communication in cultivating a safety-oriented environment. This finding is consistent with scientific investigations that highlight the significance of patient-centered care in enhancing healthcare results and patient contentment [21].

The intricate nature of medical error perceptions, which encompass divergent beliefs regarding accountability and disclosure, underscores possible domains for focused interventions. These inequalities might be rectifiable through educational initiatives that promote a collective comprehension of the reporting and disclosure procedures. This is consistent with prior studies that support the implementation of focused educational interventions aimed at improving the safety practices of healthcare practitioners in relation to patients [22,23].

The findings obtained from this study regarding the perspectives of healthcare professionals regarding the rights and safety of patients in Najran, Saudi Arabia, have substantial potential for guiding specific interventions and enhancements in the provision of healthcare. The healthcare providers' favorable disposition towards reporting medical errors indicates a possible groundwork for cultivating a strong culture of reporting within healthcare environments in Najran. The implementation of approaches designed to promote and streamline the process of error reporting may have a positive impact on ongoing quality enhancement and patient safety. The study's findings, which demonstrate contrasting perspectives on attribution and disclosure, underscore the necessity for focused educational interventions. Enhancing consensus and comprehension regarding these facets may be achieved by acknowledging the divergent viewpoints of healthcare providers; this, in turn, may promote a more uniform and transparent methodology toward error attribution and disclosure. The positive correlations observed in the study between patient rights and attitudes toward patient safety highlight the interdependence of these two vital components of healthcare. In Najran, policymakers and healthcare executives ought to contemplate the seamless integration of patient safety and rights frameworks, placing significant emphasis on the comprehensive nature of patient-centered care. Given the interrelation between patient rights and attitudes towards patient safety, fostering collaboration between healthcare disciplines is crucial. Interdisciplinary initiatives and communication channels could strengthen the alignment of patient-focused efforts, ensuring a comprehensive and integrated approach to healthcare delivery in Najran.

Although the current study provides significant contributions, it is not devoid of constraints. It is critical to acknowledge potential biases and constraints to interpret the findings in a nuanced manner. Further investigation is warranted to examine facets of the attitudes held by healthcare professionals. This may involve a thorough examination of the influence that institutional culture and individual experiences have on these perceptions. This is the first investigation of its nature to examine healthcare providers' perspectives regarding the rights and safety of patients in the Najran region. Nevertheless, there are also some limitations to this research. Since this was a cross-sectional study conducted in the Najran region, causality cannot be established, and the results may not be applicable to the general Saudi Arabian populace. It is imperative to undertake a nationwide survey involving a substantial sample size of healthcare personnel in Saudi Arabia to ascertain their perspectives on the rights and safety of patients.

## Conclusions

A considerable number of the respondents exhibited a thorough understanding of the factors that contribute to medical errors; the majority specifically expressed a solid comprehension. Providing healthcare in a safe atmosphere is of the utmost importance, as a vast majority of respondents agreed that patients had a right to access medical treatment. It is clear that patient safety and patient rights are interdependent aspects of healthcare, since there is a positive correlation between the two.

Participants' eagerness to disclose mistakes demonstrates that recognizing patients' rights is congruent with cultivating a safety culture. The findings of this study, which illustrate divergent viewpoints regarding attribution and disclosure, emphasize the need for targeted educational interventions. A more comprehensive approach to patient-centered care should be prioritized by healthcare executives and policymakers in Najran, who may think about harmonizing patient safety and rights frameworks. Future research is needed to investigate the effects of culturally tailored interventions on the attitudes and practices of healthcare providers in Najran, with respect to patient rights and safety.
